# Supplementary material for: Predictive modeling of evoked intracranial EEG response to medial temporal lobe stimulation in patients with epilepsy
Source: Commun Biol. 2024 Sep 28;7:1210. doi: 10.1038/s42003-024-06859-2 (PMC11438964; doi:10.1038/s42003-024-06859-2)
Supplement: Supplementary file 2 — Reporting Summary [file 42003_2024_6859_MOESM2_ESM.pdf]

nature portfolio

Corresponding author(s): Erfan Nozari

Last updated by author(s): Jul 19, 2024

Reporting Summary

Nature Portfolio wishes to improve the reproducibility of the work that we publish. This form provides structure for consistency and transparency in reporting. For further information on Nature Portfolio policies, see our Editorial Policies and the Editorial Policy Checklist.

Statistics

For all statistical analyses, confirm that the following items are present in the figure legend, table legend, main text, or Methods section.

n/a	Confirmed	
☐	☒	The exact sample size (*n*) for each experimental group/condition, given as a discrete number and unit of measurement
☐	☒	A statement on whether measurements were taken from distinct samples or whether the same sample was measured repeatedly
☐	☒	The statistical test(s) used AND whether they are one- or two-sided<br>*Only common tests should be described solely by name; describe more complex techniques in the Methods section.*
☒	☐	A description of all covariates tested
☐	☒	A description of any assumptions or corrections, such as tests of normality and adjustment for multiple comparisons
☐	☒	A full description of the statistical parameters including central tendency (e.g. means) or other basic estimates (e.g. regression coefficient) AND variation (e.g. standard deviation) or associated estimates of uncertainty (e.g. confidence intervals)
☐	☒	For null hypothesis testing, the test statistic (e.g. *F*, *t*, *r*) with confidence intervals, effect sizes, degrees of freedom and *P* value noted<br>*Give P values as exact values whenever suitable.*
☒	☐	For Bayesian analysis, information on the choice of priors and Markov chain Monte Carlo settings
☐	☒	For hierarchical and complex designs, identification of the appropriate level for tests and full reporting of outcomes
☐	☒	Estimates of effect sizes (e.g. Cohen's *d*, Pearson's *r*), indicating how they were calculated

Our web collection on statistics for biologists contains articles on many of the points above.

Software and code

Policy information about availability of computer code

Data collection	The work presented uses 3rd party publicly available DARPA Restoring Active Memory (RAM) dataset. No commercial software was used. RAM data can be accessed via http://memory.psych.upenn.edu/RAM
Data analysis	The data analysis was done on Python using open-source libraries and commonly known modeling algorithms. The exact methods used in our work is publicly available on GitHub: https://github.com/nozarilab/2023Acharya_evoked_ieeg

For manuscripts utilizing custom algorithms or software that are central to the research but not yet described in published literature, software must be made available to editors and reviewers. We strongly encourage code deposition in a community repository (e.g. GitHub). See the Nature Portfolio guidelines for submitting code & software for further information.

Data

Policy information about availability of data

All manuscripts must include a data availability statement. This statement should provide the following information, where applicable:

- Accession codes, unique identifiers, or web links for publicly available datasets
- A description of any restrictions on data availability
- For clinical datasets or third party data, please ensure that the statement adheres to our policy

All the data used in our work can be found in http://memory.psych.upenn.edu/RAM

Research involving human participants, their data, or biological material

Policy information about studies with human participants or human data. See also policy information about sex, gender (identity/presentation), and sexual orientation and race, ethnicity and racism.

Reporting on sex and gender	The RAM dataset consists of n=81 subjects (n=37 females and n=44 males). Out of this, we have analyzed the data of n=10 subjects randomly based on our exclusion logic. All our analysis has been done at a subject-specific level. Hence, no sex or gender based grouping was performed.
Reporting on race, ethnicity, or other socially relevant groupings	All our analysis has been done at a subject-specific level without having to group subjects based on race, ethnicity or other social groups.
Population characteristics	The RAM dataset consists of n=3 subjects under the age of 20, n=48 subjects between ages 20-40 and n=30 subjects above age 40. Out of this, we have analyzed the data of n=13 subjects randomly based on our exclusion logic. All our analysis has been done at a subject-specific level.
Recruitment	NA
Ethics oversight	NA

Note that full information on the approval of the study protocol must also be provided in the manuscript.

Field-specific reporting

Please select the one below that is the best fit for your research. If you are not sure, read the appropriate sections before making your selection.

☒ Life sciences ☐ Behavioural & social sciences ☐ Ecological, evolutionary & environmental sciences

For a reference copy of the document with all sections, see nature.com/documents/nr-reporting-summary-flat.pdf

Life sciences study design

All studies must disclose on these points even when the disclosure is negative.

Sample size	Intracranial EEG data from n=13 subjects used and each subject-specific recording has k>300 durations of electric stimulation. The sample size was constrained by the availability of data and our data exclusion logic.
Data exclusions	Only subjects with 1000Hz iEEG sampling frequency were considered for the analysis to ensure consistency.
Replication	All the results in our work is replicable. Random seeds are fixed wherever necessary to ensure reproducibility.
Randomization	NA (Our work does not use different experimental groups)
Blinding	NA (Our work does not use different experimental groups)

Reporting for specific materials, systems and methods

We require information from authors about some types of materials, experimental systems and methods used in many studies. Here, indicate whether each material, system or method listed is relevant to your study. If you are not sure if a list item applies to your research, read the appropriate section before selecting a response.

Materials & experimental systems

n/a	Involved in the study	
☒	☐	Antibodies
☒	☐	Eukaryotic cell lines
☒	☐	Palaeontology and archaeology
☒	☐	Animals and other organisms
☒	☐	Clinical data
☒	☐	Dual use research of concern
☒	☐	Plants

Methods

n/a	Involved in the study	
☒	☐	ChIP-seq
☒	☐	Flow cytometry
☒	☐	MRI-based neuroimaging

Plants

Seed stocks	NA
Novel plant genotypes	NA
Authentication	NA
